# The Cruelties of the Voting System

**Published:** 1903-09-26

**Authors:** 


### THE CRUELTIES OF THE VOTING SYSTEM.

THE evils to which we referred in our last issue under the above heading appear to be so real, and yet so little appreciated, that we quote the following notice which has been issued by the Charity Voting Reform Association :—

"The voting system is a modern invention, not known out of England, hardly out of London. It is irrational, since it ensures benefits rather to those who have influence and friends than to the friendless and forlorn. It is immoral for the voters, since they either give their votes to a particular candidate, without knowing anything of the merits of the others, or else to persons of whom they know nothing. Also immoral for the candidates, to whom it serves as a school of mendicancy, and to whom it occasions expenses which they can ill afford, and which they should not have to incur. It also involves loss of time in canvassing, and frequent disappointment. It is dishonest, since it encourages bartering for votes, trickery, and false promises. It is cruel, since from three to ten times the number of candidates for whom there are vacancies being placed on the lists, the unsuccessful majority are left to repeat the weary process, till, often in cases of children, they are past the age, and in cases of old and sick people their candidature is cut short by death. It is the duty of managers of institutions to have the cases of all the applicants thoroughly investigated, and their relative merits seriously compared, with some regard, if possible, to priority of application. The result of these processes should determine the selection of the candidates. Thousands of persons, quite unconnected with each other cannot possibly perform these processes, and until voting is formally abolished it will be well for subscribers to place their proxies in the hands of the committees to be administered. The committees should at the same time be urged to invite all subscribers to follow the same course, and, in fact, several societies already adopt this course. Further, subscribers should insist that the number of candidates should never exceed double that of the vacancies. Should the committees refuse to adopt these improvements, there is only one course open to the benevolent, namely, to refuse further subscriptions, and to transfer their aid to other charities where the objectionable system of voting is not in force."

THE Capetown Legislative Council has decided to ask the Government to consider the desirability of extending parliamentary franchise to spinsters and widows.

